# COVID-19-Associated Acute Psychotic Disorder—Longitudinal Case Report and Brief Review of Literature

**DOI:** 10.3390/medicina59020408

**Published:** 2023-02-19

**Authors:** Maria Gabriela Puiu, Vlad Dionisie, Andra Ioana Dobrin, Mirela Manea

**Affiliations:** 1 Department of Psychiatry and Psychology, “Carol Davila” University of Medicine and Pharmacy, 020021 Bucharest, Romania; 2 “Prof. Dr. Alexandru Obregia” Clinical Hospital of Psychiatry, 041914 Bucharest, Romania

**Keywords:** COVID-19, coronavirus disease-19, psychotic disorder, psychosis, SARS-CoV-2

## Abstract

Even though since the beginning of the COVID-19 pandemic, the literature became more and more abundant on data and hypotheses about the various consequences on people’s lives, more clarity needs to be added to the existing information. Besides the stressful experiences related to the COVID-19 pandemic, SARS-CoV-2 infection has been proven to impact brain functioning through direct and indirect pathogenic mechanisms. In this context, we report a case of a patient presenting with a first episode of psychosis following COVID-19. In our case, a 28-year-old male patient with no personal or family psychiatric history developed psychotic symptoms (delusions, hallucinations, and disorganized behaviour) that required antipsychotic treatment and inpatient hospitalization one week after he was discharged from the hospital after COVID-19. At the six-month and one-year follow-up, the patient was in remission without any psychotic signs or symptoms. A brief review of the literature is also provided. The case presented in this article outlines the possibility that the post-COVD-19 recovery period might be a crucial time for the onset of acute psychotic disorder, and therefore, routine psychiatric assessments should be carried out during all phases of the disease. A clearer picture of the impact of the COVID-19 pandemic on mental health will most likely be revealed in the future as many consequences need long-term evaluation.

## 1. Introduction

The World Health Organization declared coronavirus disease-19 (COVID-19) as a global pandemic in March 2020 [1]. Aggressive restrictive measures were adopted worldwide in order to mitigate the rapid spread of the severe acute respiratory syndrome coronavirus 2 (SARS-CoV-2), which is responsible for COVID-19 [2]. Therefore, besides its direct impact on health, the COVID-19 pandemic exerted a huge impact on the environment, economy, education, and human psychology [3], leading to serious societal changes that were unprecedented for almost all people. It can be said that no individual remained untouched by the pandemic. The impact of the COVID-19 pandemic on mental health may be related to one or more of the following: the social impact of the pandemic and of the containment measures [4,5], the fearful experiences associated with direct and indirect perceived consequences of the COVID-19 pandemic [6,7,8,9,10], and the direct and secondary consequences of the SARS-CoV-2 infection in the brain [11]. It was demonstrated that SARS-CoV-2 can enter the central nervous system (CNS) by hematogenous route via the blood–brain barrier (BBB) or by retrograde transport using infected peripheral nerves (olfactory invasion) [11]. Moreover, the “cytokine storm” and hypoxia seen in COVID-19 patients can lead to the disruption of the BBB and subsequent brain inflammatory damage [12]. All of these mechanisms may be involved in various degrees in the development of psychiatric disorders in COVID-19 patients.

Since it is a multifaced problem, the mental health burden of the COVID-19 pandemic should be approached from at least two angles: one related to a possible increase in the incidence and prevalence of certain psychiatric disorders (e.g., depressive disorders, anxiety disorders, and psychosis) in the general population due to the pandemic and one related to the possible medium- and long-term psychiatric complications of this disease. Concerns regarding the onset of stress related psychiatric symptoms have been raised since the beginning of the pandemic and, later on, were supported by research that showed high levels of distress, anxiety, and depression in the general population during the COVID-19 pandemic in general [13] but also during specific interventions such as the lockdown [14] and quarantine [15,16,17]. These results reveal that the COVID-19 pandemic is an important stressor that greatly contributes to the onset of a variety of mental health problems. Some post-acute COVID psychiatric symptoms, such as fatigue, cognitive impairment, anxiety, and depression, have been extensively studied, which has resulted in a compelling bulk of evidence [18]. Conversely, little data is available regarding psychotic symptoms but the number of case reports describing new onset psychosis related to the COVID-19 infection has constantly increased since the beginning of the pandemic, thus paving the way for gaining more knowledge about this condition.

In this article, we present a new case of a patient with acute psychotic disorder induced by COVID-19 requiring antipsychotic treatment and inpatient hospitalisation with the aim to provide additional insight into this emerging consequence.

## 2. Case Presentation

In March 2021, a 28-year-old male patient presenting with rhinorrhoea and fatigue was diagnosed with SARS-CoV-2 infection by real time-polymerase chain reaction (RT-PCR) test and initially received outpatient follow-up and treatment (acetaminophen 500 mg per os—p.o., twice daily) for 7 days from his general practitioner. His condition started to worsen; therefore, he was admitted to “Marius Nasta” National Institute of Pneumology for dry cough, progressively aggravated dyspnoea, diarrhoea, rhinorrhoea, and an oxygen saturation of 86% in room air.

The patient denied any personal psychiatric or medical history as well as any family psychiatric history. The collateral history provided by his family confirmed this information. He was living with his mother, had never married, and had no children. He had completed secondary education and was a small business owner. He denied using tobacco or any other drug and alcohol abuse. Overall, the patient had a good premorbid adjustment.

During the 14 days of hospitalization in the pneumology hospital, the patient was prescribed antiviral treatment with remdesivir intravenously (i.v.) for five days (200 mg for the first day and then 100 mg/day for the rest of the days), corticosteroid treatment with dexamethasone (16 mg i.v. daily), low-molecular-weight heparin (LMWH) (10,000 IU subcutaneously daily), anti-inflammatory treatment tocilizumab i.v. (single dose of 800 mg), omeprazole (20 mg p.o. daily), and oxygen therapy via nasal cannula with a flow of 12 L/min, which was progressively decreased due to his improving condition. The patient did not require intensive care unit admission. The oxygen therapy was stopped 24 h before discharge. He was discharged from the hospital in a good general condition and with at-home treatment recommendations that consisted of a decreasing regimen of dexamethasone (16 mg/day for 7 days then 8 mg/day for another 7 days), omeprazole (20 mg/day for 14 days), and acetylsalicylic acid (75 mg/day for 1 month). The patient was discharged with the diagnosis of severe bilateral viral pneumonia due to SARS-CoV-2 infection with respiratory hypoxemic failure and obesity (class 1). No psychotic symptoms or abnormal/bizarre behaviour were noted by the medical personnel during hospitalization in the pneumology hospital.

A week after the patient was discharged from the pneumology hospital, he was brought to the psychiatric emergency department of “Prof. Dr. Alexandru Obregia” Clinical Hospital of Psychiatry by ambulance and police. The patient was admitted for florid psychotic symptomatology, which consisted of visual, visceral, and auditory hallucinations (hearing the voice of God talking to him, receiving signs from God, seeing parts of demonic bodies, and feeling demons inside his body that attempted to possess him to take him to hell), delusional ideas (the patient claimed that the apocalypse will come soon and his loved ones together with the entire world will suffer; the patient also claimed that he had a special mission given by God, precisely to pray continuously in order to save himself and his loved ones), bizarre and aggressive behaviour consistent with hallucinations and delusions (praying out loud in the middle of the room or hallway; responding verbally to unseen stimuli, most probably to auditory hallucinations; keeping his eyes closed because God told him to do so), and psychomotor agitation. The mental state examination also revealed that the patient was conscious; fully oriented in person, place, and time; very anxious; and denied any suicidal thoughts. Given that the patient was very uncooperative, the presence of severe psychotic symptomatology that was causing distress and changes in behaviour, and also the refusal of being admitted, the patient was hospitalized under compulsory admission.

The modified overt aggression scale (MOAS) score (on the first day of admission) was: verbal aggression, 2 points; physical aggression towards others, 2 points. The Positive and Negative Symptoms Scale (PANSS) score (on the second day of admission) was: total, 111; positive symptoms, 29; negative symptoms, 19; general psychopathology, 63. The Confusion Assessment Method (CAM) screening was negative on all daily assessments.

For the following days of admission, the symptomatology persisted but the patient showed continuous improvement of symptoms, and complete resolution of psychosis was achieved by discharge. PANSS score (at discharge) was: total, 43; positive, 10; negative, 11; general psychopathology, 22.

The collateral history provided by his mother and sister revealed that, a couple of days after the patient was discharged from the pulmonology hospital, he started hearing voices described as God’s voice, became increasingly agitated, had sleeping difficulties, and was very anxious.

The physical examination on admission to the psychiatric department revealed hepatomegaly and class 1 obesity (body mass index = 33.24 kg/m^2^). On admission, his vital signs were the following: heart rate, 92 per minute; blood pressure, 125/80 mmHg; and respiratory rate, 13 breaths per minute with an oxygen saturation of 98% in room air. No COVID-19-related signs or symptoms were presented at this point, except for mild fatigue. Haematological (complete blood count), biochemical (metabolic profile and hepatic and renal panels), and toxicological tests were performed and revealed slightly elevated values for: gamma glutamyl transpeptidase, 141 U/L (reference 12–64 U/L); aspartate aminotransferase, 59 U/L (reference 5–34 U/L); alanine aminotransferase, 144 U/L (reference 5–55 U/L); and triglycerides, 165 mg/dl (reference 0–150 mg/dL); six days later, the value of transaminases were still abnormal: gamma glutamyl transpeptidase, 106 U/L; aspartate aminotransferase, 50 U/L; and alanine aminotransferase, 162 U/L. The patient was screened for viral hepatitis B and C and was found negative. The hepatomegaly was considered to be due to the treatment with remdesivir and tocilizumab [19]. Full neurological examination was normal. A computed tomography (CT) of the brain without contrast was performed and the result showed mild parietal bilateral cortical atrophy. Electrocardiogram (ECG) and electroencephalogram (EEG) were unremarkable. COVID-19 rapid antigen test was found negative. After discharge from the pneumology hospital, the patient did not follow the prescribed treatment of dexamethasone, omeprazole, and acetylsalicylic acid.

Initially, the patient was treated with haloperidol 5 mg intramuscular (i.m.) twice daily and diazepam 10 mg i.m. twice daily due to refusal of oral medication, psychomotor agitation, and aggressiveness. After two days, the patient became more cooperative and calmer and accepted oral treatment; therefore, he was switched to aripiprazole up titrated to 15 mg p.o. daily and divalproex sodium 1500 mg p.o. daily for the rest of his hospital stay. The patient had a good clinical response to the antipsychotic treatment with full resolution of the symptoms and developed insight into illness. He was discharged 12 days later with the diagnosis of acute and transient psychotic disorder.

The diagnosis of delirium was rejected since the patient did not have alterations in the level of consciousness or attention during daily evaluations and also screened negative on all repeated assessments using CAM. Further, corticosteroid treatment-induced psychosis was considered in the differential diagnosis even though hypomania or mania are the most common psychiatric symptoms as a consequence of corticosteroids. This diagnosis was excluded since the symptoms’ onset is typically in the first 1–2 weeks of treatment while our patient developed the psychotic symptomatology later and despite the interruption of the steroid treatment [20], and the total dexamethasone dose was low. However, we could not definitely exclude the possible additive role of corticosteroids in the onset of psychotic symptoms in a susceptible patient. Recent research has not shown a link between remdesivir treatment and any neuropsychiatric symptoms [21]. Further, tocilizumab was not observed to induce psychosis-like symptoms in COVID-19 patients, and, in addition, it was studied as an add-on treatment in schizophrenia [22,23]. Tocilizumab and remdesivir do not have a significant interaction with the metabolism of dexamethasone (i.e., raising or lowering blood levels of dexamethasone) [24]. First-episode schizophrenia has been suggested as an explanation but, in this case, the patient had a good psychosocial functioning before the psychotic episode and the episode had a sudden onset with no previous signs or symptoms of chronic psychosis or prodromal phase. Therefore, taking into consideration all of the above and, in particular, the specific onset (i.e., after COVID-19) and clinical presentation, an aetiological relationship between the psychotic symptoms and COVID-19 was presumed. The possible mechanisms taken into consideration involved the psychological stress factors related to COVID-19, the multiple immunological effects on the central nervous system (CNS) of COVID-19, and, to a lesser extent, the adverse reactions to the COVID-19 treatment. Accumulating evidence supports the putative role of all these causes in the pathophysiology of COVID-19-associated psychosis [25].

After discharge from the hospital, the patient continued his psychiatric treatment for three months but then he decided to fully withdraw the treatment against medical advice. He resumed his job and he was without any signs or symptoms of lingering psychosis at the six-month and 1-year follow-up after discharge.

## 3. Discussion and Brief Review of Literature

In this paper, we report a novel case of a patient presenting with a first episode of psychosis following COVID-19. The case describes a male individual with no personal or family psychiatric background who developed positive psychotic symptoms of auditory hallucinations, delusional ideas, and disorganised behaviour soon after he was discharged from hospital after COVID-19. The episode was completely resolved with two weeks of antipsychotic treatment. The longitudinal presentation of this case, which includes follow-up information at 6 months and 1 year after, differentiates our case.

Since the early stage of the pandemic, cases describing patients with COVID-19-induced psychosis were increasingly reported in the literature with the aim to highlight one of the numerous possible complications of this rapidly emerging infectious disease. Concerning psychopathological features, numerous cases presented female or male individuals with different types of delusions (i.e., persecutory, grandeur, reference, death, and religious) and auditory hallucinations [26,27,28,29,30,31,32,33,34,35,36,37,38,39,40,41] as the main part of clinical presentation. Of note, similar to our case, the symptomatology reported in some patients included visual or tactile hallucinations as well [28,31,32,33], which are a common characteristic of psychosis due to organic aetiology [42]. These particular COVID-19 associated symptoms could be explained by the hypothesis of SARS-CoV-2-induced brain injury, which is thought to be determined through direct neuronal invasion and systemic massive inflammation [20,43]. Cotard’s syndrome, typically characterised by nihilistic delusions and associated with perceived life-threatening illnesses [44], was observed in some cases [31,45,46]. Other reported clinical features of COVID-19-induced psychotic episodes included suicidal behaviour or ideation, aggressive or disorganised behaviour (which is similar to our case), and catatonia [29,30,32,35,38,47,48,49]. Even though numerous case studies have provided a detailed clinical presentation of the patients, assessment of symptom severity using rating scales is an important aspect that should not be overlooked in order to objectively describe the extent of the clinical picture. To the best of our knowledge and similar to our case, only Gorkcay et al. (2022) [41] and Gullulu et al. (2022) [50] have reported the total PANSS score at admission as well as at discharge. Under antipsychotic treatment with aripiprazole or haloperidol and chlorpromazine, the patients’ total PANSS scores dropped from 132 to 48 points [41] and from 131 to 42 points [50], respectively. In our case, the patient had a total PANSS score at admission of 111 points and at discharge of 43 points.

In terms of the timeframe between COVID-19 and the development of psychotic symptoms, various cases showed that psychiatric symptoms appeared during the acute stage of the disease [30,40,41], while some cases, including the one reported herein, presented after the resolution of illness [27,47,51] or during asymptomatic or mild COVID-19 [26,32,36,39]. The latter might particularly add to the supporting evidence of either subacute sequelae caused by SARS-CoV-2 neuroinvasion or COVID-19-related stress trigger since there is no evidence of massive systemic inflammation. These data showed that a possible psychotic episode can occur during all stages and regardless of the severity of the disease; therefore, a watchful waiting approach would be ideal for monitoring the mental health of COVID-19 patients.

The cornerstone of pharmacological treatment was represented by different low-to-moderate dose of antipsychotics, such as risperidone, paliperidone, olanzapine, aripiprazole, ziprasidone, and haloperidol. Additional psychiatric drugs included benzodiazepines (i.e., lorazepam or diazepam) and valproic acid [30,31,32,35,38,39,40,46,47]. In some cases, and in ours as well, intramuscular administration of benzodiazepines and/or haloperidol was used for the rapid management of anxiety and behavioural disturbances [28,37,40,45]. Electroconvulsive therapy was initiated in cases unresponsive to pharmacological treatment [52,53].

Of all diagnostic tests performed to identify the possible SARS-CoV-2 direct brain involvement, RT-PCR testing for SARS-CoV-2 in the cerebrospinal fluid (CSF) could be of great importance in the differential diagnosis step. Elfil et al. (2021) [49] and Austgen et al. (2022) [53] performed RT-PCR test of CSF samples of their patients and reported negative results. This is only to be expected, since a recent systematic review of cases with positive detection of SARS-CoV-2 RNA in the CSF identified only patients with neurological diagnosis, most commonly encephalitis [54]. Gullulu et al. (2022) [50] reported a patient with SARS-CoV-2 antibodies in the CSF. Apart from C-reactive protein, a non-specific marker of inflammation which is increased in COVID-19, interleukin (IL)-6 is another laboratory test performed in order to confirm systemic inflammation. Austgen et al. (2022) [53] and Łoś et al. (2021) [40] reported increased levels of IL-6 in their patients (17 pg/mL and 79.1 pg/mL, respectively) which could link a certain neuroinflammation pathway to the occurrence of psychosis.

Evidence coming from large cohort studies (n = 1,284,437 and n = 236,379 adults, respectively) indicates that patients had an increased risk for psychotic disorders in the follow-up period of 6 months and also of 2 years after COVID-19. Conversely, authors concluded that the increased incidence for depressive and anxiety disorders was transient [55,56].

Timely follow-up is essential for improving treatment adherence, achieving remission, and also for monitoring possible diagnostic shifts over time since the outcome of first-episode psychosis is highly variable [57,58,59]. Numerous studies indicated that individuals with acute and transient psychotic disorders had a low temporal diagnostic stability at the 1-year or 2-year follow-up. An increased number of these patients had a diagnostic shift to schizophrenia [60,61]. Thus, these data reinforce the need to follow-up patients with COVID-19-induced psychosis after the initial episode. Several case studies reported that patients were asymptomatic at three or six months after COVID-associated psychosis [39,50,51,62,63]. Kazi et al. (2021) [28] reported a patient who received a diagnosis of “bipolar disorder manic episode with psychosis” eight months after COVID-19-induced psychosis. In our case, at the 6-month and 1-year follow-up, the patient was in complete remission without any evidence of chronic psychosis. In view of current concerns regarding an increased risk of schizophrenia following COVID-19 [64], future follow-up cohort studies are highly needed.

Delirium is a clinical syndrome that can be characterized by psychotic signs and symptoms with an acute onset and has various aetiologies, including COVID-19 [65]. Therefore, it is necessarily to be considered in the differential diagnosis of acute psychosis in patients with SARS-CoV-2 infection. Two systematic reviews of case reports of COVID-19-induced psychosis highlighted that more than half of the analysed case reports did not include delirium in the differential diagnosis. Moreover, 27% of the reviewed reports that considered delirium did not mention a formal assessment [66,67]. In the case documented in this paper, delirium was excluded based on clinical judgement and repeated negative screening results using CAM.

## 4. Conclusions

The case presented herein illustrates once again the important role played by COVID-19 in the onset of acute psychotic disorders and outlines the possibility that post-COVID-19 recovery period might be a crucial time for the onset of acute psychotic disorders. Psychiatric assessments should be routinely included in the standard of care for COVID-19 patients during all phases of the disease (from diagnosis to after resolution) and specific interdisciplinary guidelines are required. Raising awareness of the appearance of post-acute COVID psychiatric complications, such as psychotic disorders, will contribute to improving personalised diagnosis, follow-up, and overall management of patients.

## Data Availability

The data presented in this study are available on reasonable request from the corresponding author. The data are not publicly available due to ethical and institutional reasons.
